# An Introspective Update on the Influence of miRNAs in Breast Carcinoma and Neuroblastoma Chemoresistance

**DOI:** 10.1155/2014/743050

**Published:** 2014-12-04

**Authors:** Alessia Carta, Rachel Chetcuti, Duncan Ayers

**Affiliations:** ^1^Faculty of Health Sciences, University of Malta, Msida MSD 2080, Malta; ^2^Department of Pathology, Faculty of Medicine and Surgery, University of Malta, Msida MSD 2080, Malta; ^3^Faculty of Medical and Human Sciences, The University of Manchester, Manchester M1 7DN, UK

## Abstract

Chemoresistance to conventional cytotoxic drugs may occur in any type of cancer and this can either be inherent or develop through time. Studies have linked this acquired resistance to the abnormal expression of microRNAs (miRNAs) that normally silence genes. At abnormal levels, miRNAs can either gain ability to silence tumour suppressor genes or else lose ability to silence oncogenes. miRNAs can also affect pathways that are involved in drug metabolism, such as drug efflux pumps, resulting in a resistant phenotype. The scope of this review is to provide an introspective analysis on the specific niches of breast carcinoma and neuroblastoma research.

## 1. Introduction

Carcinogenesis is a dynamic process in the cell genome involving multifactorial steps [1]. Six distinct changes are observed in general malignant growth that drives the progressive transformation of normal human cells into invasive cells [2]. The onset of this internal potential for self-proliferation is the result of the mutation of particular genes that are responsible of cell growth [3, 4]. The alteration in the normal genetic material consequently causes errors in the cell cycle. This induction occurs as the genes that are mutated encode proteins that usually progress the cell cycle orderly [3].

Cancer develops as the independent growth is insensitive to antigrowth factors or environmental signals. CD44 cell surface molecule, normally involved in the cell-to-cell interaction [5], is evidently observed to decrease in neuroblastoma development [6]. The results of impaired cell motility are inability of induction of apoptosis and cell cycle arrest. Collectively these features lead to limitless replication potential followed by the intrinsic ability to encourage angiogenesis [7].

## 2. Breast Cancer Development

Similar to other solid tumours, development of breast cancer occurs through a combination of epigenetic changes and the molecular aberrations mentioned earlier [8]. Breast cancer is the most commonly diagnosed malignancy and the major cause of death amongst women worldwide [9]. It is considered to be a highly heterogeneous disease [10] since it may develop due to varying molecular features and clinically presents itself in diverse manners [11]. Two major gene classes are crucially linked with the development of breast cancer and are described below.

### 2.1. HER-2 Gene

Tyrosine kinases are proteins which serve as mediators of cell signaling pathways, affecting both cell survival and growth [12]. A classical type of these receptor tyrosine kinases is the erythroblastic leukemia viral oncogene homolog (ErbB) family of receptors, which includes four subfamilies: the ErbB or the human epidermal growth factor receptors (HER-1 to HER4, or ErbB-1 to ErB-4) [13]. All of these receptors are crucial for orchestrating the proliferation and differentiation of normal cells and can therefore regulate growth [14]. Each receptor is composed of three structural domains: a cytoplasmic area for tyrosine kinase receptor, a transmembrane domain, and an extracellular binding site for ligands [15]. Mutations in this family of receptors tend to be of a somatic type, such that the abnormality occurs after conception, rather than passed on through the family [16].

Observably, anomalous activity of a particular receptor within this family, HER-2, has been identified in approximately 20–30% of breast cancer cases [8, 13]. This receptor is encoded by the HER-2 protooncogene located on chromosome 17q [14]. Growth factor receptors become activated upon ligand binding and receptor dimerization [13]. The latter can either be heterodimerization, which occurs between two receptors of the same family, or homodimerization, which occurs between two identical receptors [17]. This dimerization process helps to activate the tyrosine kinase segment of the receptor which results in the phosphorylation of multiple tyrosine residues [15]. These create multiple transduction pathways acting on downstream receptor proteins which finally cause the physiologic responses that had been previously signaled, such as cell division and apoptosis [17]. Such pathways include the 3-kinase (PI3K)/Akt pathways and mitogen-activated protein (MAP) kinase [8].

This process is often dysregulated in breast cancer, where amplification of the HER-2 protooncogene results in overexpression of the receptor which fuels excessive cellular proliferation and invasiveness and can also affect drug resistance pathways [13]. In essence, genetic anomalies alter the HER-2 protooncogene into an oncogene through a gain-of-function role that stimulates excessive cellular division, creating disproportionate cellular growth. The mutations causing this type of oncogenic change are usually of the dominant type, such that a mutation in just one allele may disrupt the whole reproductive program of the cells [18].

### 2.2. BRCA Genes

Besides abnormalities in protooncogenes, the genes that examine the cell cycle at specified checkpoints can be mutated and result in carcinogenesis. Mutations in these tumour suppressors are found in a spectrum of cancer models, including breast cancer. A particular set of tumour suppressors associated with breast cancer is the Breast Cancer 1 and 2 gene set (BRCA1 and BRCA2) [19]. Similar to other tumour suppressors, the product expressed from this family of genes helps to mediate the rate of the cell cycle [20]. Various studies indicated that germ-line mutations in these genes were linked to an increased chance of developing breast cancer [21–24]. In 1990, DNA linkage studies testing family members with similar features, such as early onset of breast cancer and familial history of both breast and ovarian cancers, were carried out [22]. The first gene found to be abnormally expressed was the BRCA1, located on chromosome 17 [22]. Further studies continued until four years later, when a similar gene on chromosome 13 was also found to be mutated in patients presenting with similar clinical symptoms and, consequently, this novel gene was named BRCA2 [22]. In addition, both mutations in these genes are inherited in an autosomal dominant pattern [22].

Both of these genes code for proteins which are responsible for the regulation of genomic stability, ranging from DNA damage response and repair to apoptosis [25]. BRCA1 gene is related to having a specific role in regulating transcription, cell cycle, and DNA damage [25]. In a study carried out by Scully et al., this association has been supported since the BRCA1 resultant protein can interact with the basal transcriptional machinery of a cell, including RNA polymerase II and transcription factors [26]. The role of BRCA1 protein as a tumour suppressor became even more evident with its role in the intensification of the centrosome and at the G_2_/M checkpoints of the cell cycle [27]. The centrosome is a vital organelle which regulates the association of microtubules which helps maintain cell structure and polarity, coordinating the development of the mitotic spindle [28]. Specifically, Hsu and White had validated that the BRCA1 tumour suppressor can form a link with *γ*-tubulin, a critical protein within the centrosome [27]. Its association with *γ*-tubulin and the centrosome confers evidence that the BRCA1 plays a crucial role in the orderly regulation of the mitotic spindle formation and the G_2_/M checkpoint [29]. Consequently, suppressed BRCA1 expression, which commonly occurs in cancer cells, results in abnormally hastened cellular proliferation. This has been verified in a study by Thompson et al., where aggressive cellular proliferation of mammary cells was noted after experimentally inhibiting BRCA1 expression with the use of antisense oligonucleotides [30].

Nonetheless, despite the variation of their protein sequence, both BRCA1 and BRCA2 have mutual biological roles [31]. Both genes have significant presence in both mitotic and meiotic cells [32], where the proteins expressed by these genes are related to Human RAD51 [33]. It has been ominously found that this has a fundamental role in homologous recombination and DNA double-stranded break repair [34, 35]. Various studies validated that the messenger RNA (mRNA) expression of both BRCA1 and BRCA2 genes escalates as the cell moves into S phase, ascertaining that their biological functions culminate during (or after) DNA replication [36–38]. This protein, Human RAD51, is vital in homologous recombination [25] and in repairing double-stranded DNA breaks by amalgamating single-stranded DNA, resulting in a nucleoprotein filament which has the potential to occupy a homologous duplex DNA molecule [39]. This provides evidence that both BRCA1 and BRCA2 assist in a damage response pathway. Consequently, aberrant functional behavior silencing the roles of BRCA1 and BRCA2 will result in irregular DNA structures.

Considering that breast cancer is claimed to be a heterogeneous disease, an association between both environmental and genetic elements definitely has a role in its formation [25]. Although mutations in HER-2 and BRCA genes tend to be the most commonly studied abnormalities, other genes and receptors have also been found to induce breast cancer. These mainly include the tumour protein 53, phosphatase and tensin homolog gene, and the mitogen-activated protein kinase kinase kinase-1 which is associated with a slimmer chance to induce breast cancer [40, 41].

### 2.3. Clinical Presentation

Initially, breast cancer exhibits no symptoms as the growth might be too small and undetectable, yet highly curable. Patients usually present to a clinician after a physical, painless lump is detected around the breast area. In less frequent cases, the presence of a lump is accompanied by breast pain or noticeable changes in the breast, such as redness, abnormal nipple discharge such as blood, or sensitive tenderness [42]. Diagnostic tests are initially noninvasive, where the contour of the lump can be felt either by palpable examination or by radiographic methods such as mammography or ultrasound. At this point, the clinician must deduce whether the lump is benign or if a malignancy is suspected. In queried cases, a biopsy from the breast is collected via needle or surgical incision, where microscopic analysis of the tissue can establish the nature of the growth [43]. Apart from an initial diagnosis, microscopic results also establish the extent of the malignancy and the pattern of the growth. In cases resulting as positive for breast cancer, further tests are carried out to identify the type of malignancy [43].

A breast malignancy can start developing either in the ducts or in the lobules, thereby termed as a ductal or lobular breast carcinoma, respectively (DCIS or LCIS), and in less common cases from the stromal tissues of the breast [44]. Each type of breast cancer has been classified throughout the years by gene expression profiling which classifies breast cancer into different molecular subtypes, as explained in Table 1.

Molecular markers are recently being highly utilized to deduce the presence or absence of estrogen receptors, progesterone receptors, and human epidermal growth factor receptor in breast cancer [45].

The subdivision of the type of malignancy is critically important when prescribing treatment, as one therapeutic measure can be highly effective for one breast cancer subtype, yet barely effective in other subtypes [45].

### 2.4. Breast Cancer Therapies

In 2012, 1.7 million cases of breast cancer were diagnosed on a global scale [46]. It is believed that one-third of breast cancer mortality rates can be reduced through earlier malignancy screening implementation and therefore allowing for more prompt optimal treatment initiation [47].

The selected treatment is primarily based on the staging and/or grading of the phenotypic characteristics of the malignancy [48]. Consequently, accurate microscopic screening has a significant effect on the outcome of the patient as the staging determines the treatment protocol. Other factors such as risks and benefits, age of the patient, and the patient's predicted compliance to the proposed therapy also influence treatment decisions [48].

Staging of an individual tumour depends on the characteristics observed during clinical examination or else during the pathological stage that deals with the features observed after surgery [49]. Pathological staging is the most commonly implemented measure as it gives a more accurate prognosis [49]. International criteria staging the features of the tumour cells are utilized worldwide, following the stages indicated by the American Joint Committee on Cancer (AJCC) TNM system [49]. The staging levels are concluded by combining the tumour size (T), the degree of metastasis into lymph nodes neighbouring the breast (N), and metastasis into other organs (M) [49].

Histological grading is the overall score established upon microscopic evaluation of both morphological and cytological features of the cancerous cells [50]. The grading provides information regarding the prognosis while staging sheds light on the overall progression of the cancer.

Depending on the tumour grade/stage and the molecular characteristics of the malignancy, treatments vary, ranging from surgery, chemotherapy, radiation, hormonal, and targeted therapy [51]. Chemotherapy is the most commonly used treatment where it is also utilised with other therapies and even after surgery [10]. The simultaneous employment of multiple therapeutic measures has effectively improved the rate of breast cancer survival. Nonetheless, a completely effective response to chemotherapy is still highly limited in the majority of cases [52]. Consequently, it is noted that initially most of the patients respond effectively to chemotherapy, though this response is highly reduced after a set drug exposure time period that varies between individual patients. Furthermore, various studies demonstrated that a subgroup of patients is inclined [53, 54]. One study in particular carried out by Brewster et al. confirmed that breast cancer recurrence rates were 11% after 5 years and 20% after 10 years of therapy [54]. This variation in treatment response is believed to be in part due to the tumour developing molecular mechanisms of resistance to chemotherapeutic drugs [55].

## 3. Neuroblastoma

Neuroblastoma is an aggressive tumour growth of the primordial cells of the sympathetic nervous system. It is the most common solid extracranial neoplasm embryonal tumour [56]. This growth arises during foetal or early postnatal life and is remarkable for its spontaneous regression or maturation, regardless of the treatment [57, 58]. This disease heterogeneity can display a very aggressive, malignant phenotype that is weakly sensitive to present intensive multimodal therapy [57]. Neuroblastoma presents with dynamic genetic variations, as exhibited in most human cancer developments, which influences the combination of the therapy given to the patient [59]. The degree of aggressiveness and risk of regression of neuroblastoma are sorted into groups for better classification [60]. The risk stratification, age, and the complex genetic pattern of the disease collectively contribute to the regression or progression of this malignancy [56–58].

### 3.1. Incidence

Neuroblastoma presents approximately 100 new cases per year in UK as quoted from The Neuroblastoma Society (UK) [61]. Evidence in the research by the* SEER Cancer Statistics*, based in USA, indicates that the incidence rate of neuroblastoma in children less than a year old is of 51.5 per 1,000,000 subjects. This study was age-adjusted to the 2000 US Std Population (19 age groups, Census P25-1130), from which patients being of age one or less add to 40% of all the diagnosed cases [62]. The median age of diagnosis of neuroblastoma is of 18-19 months [58, 59, 63] where it represents 8% of all malignancies diagnosed in paediatric patients younger than 15 years [64]. Furthermore, in more recent data neuroblastoma was the causal death of 15% of the diagnosed patients in 2006 [65].

### 3.2. Clinical Presentation

Most neuroblastoma tumours present with undifferentiated sympathetic nerve cells arising from the neural crest [57]. Presenting signs and symptoms are highly variable including fatigue, unexpected fever, weight loss, irritability, and bone pain [66]. Furthermore, signs vary depending on the site at which this primary tumour occurs and also whether metastasis had developed or not [66]. In most cases of primary tumour, the patient presents malignant growth in the abdomen region, where at least half of these arise from the adrenal medulla. Next common sites involve the neck, chest, and pelvis [58]. Around 40% of the patients present with localized disease, albeit in majority of neuroblastoma cases metastasis to the lymph nodes, bone marrow, bone, liver, and skin occurs [58]. With a small percentage of 5% of the patients, a striking phenotype of 4S disease is observed, where these infants develop tumours that metastasize to skin, liver, and bone marrow (<10% invasion). 4S tumours tend to disappear spontaneously without treatment [62].

The tumour original site is not an independent prognostic factor. The presence of typical histopathological features, presence of tumour cells in a bone marrow aspirate/biopsy, and raised concentration of urinary catecholamines further conclude the development of neuroblastoma growth [58].

Cell-surface marker CD44, involved in cell-cell and cell-matrix interactions, can be used as a diagnostic marker for the aggressiveness of cancer [5]. In low risk patients, the CD44 is found expressed and thus enhanced the theory that with the absence of N-myc amplification there is a favourable clinical outcome [5].

### 3.3. Staging

Modifications of this latter classification system reflected a need to classify neuroblastoma presurgically. The International Neuroblastoma Risk Group (INRG) task force developed a consensus for pretreatment risk stratification of children with neuroblastoma. Cooperative groups of paediatricians met in 2005 to work on classifying the tumour based on imaging and bone marrow morphology rather than the extent of surgical resection. The chart was obtained by a study involving 11054 patients worldwide [60]. Stages L1 and L2 distinguish the regional tumours from involving just local structures (L1) and from invading locally (L2).

The two major important clinical variables of neuroblastoma are the age and the stage at diagnosis [67]. In all the stages of the disease higher than stage one, children of age younger than 12 months have had significantly better disease-free survival rates than older children with equivalent stages of disease [60]. Monclair et al., on behalf of the INRG, suggest that an age-at-diagnosis cut-off of greater than 18 months is associated with greater risk of disease recurrence [60]. Another crucial point in staging and recurrence is the genetic makeup of the developed neuroblastoma.

### 3.4. Genetic Aspects

Molecular abnormalities with regard to neuroblastoma involve multiple genetic aberrations that give rise to the heterogeneous features of this malignancy. Aggressiveness of the cancer phenotype can be related to loss of tumour suppressor function that is an effect of loss of genetic material or mutation in the responsive gene [4, 56]. Chromosomal abnormalities, in particular those involving protooncogenes, are also responsible for uncontrolled growth leading to the gain of protein function forcing cells to enter cell cycle [4, 68]. The four majorly discussed genetic changes in neuroblastoma are MYCN protooncogene amplification, DNA content, and the allelic deletions on chromosomes 1p and 11q or the gain of 17q [62, 68, 69]. In addition, activating mutation of the tyrosine kinase ALK (anaplastic lymphoma kinase) was recently discovered in both familial and sporadic neuroblastomas [70]. Studies showed familial neuroblastoma displayed rarely, about 1-2% of the patients with the incomplete inheritance of the autosomal dominant pattern [70]. In such cases, this presents at an early age with bilateral adrenal or multifocal primary tumours.

#### 3.4.1. MYCN Amplification

The* myc* gene alteration is involved persistently in various types of cancerous growth and is regarded with the expression of multiple genes that encode regulatory proteins [2]. Within this family of transcriptional factors, two particular proteins are expressed by N-myc gene section [71]. The role of this MYCN (N-myc) gene in oncogenesis is thought to involve extrachromosomal elements [72] or intrachromosomal homogeneously staining regions [68]. A finding by Charron et al. concluded that the regulation of the spatiotemporal gene expression of N-myc during the development significantly regulated DNA elements [73]. The amplification of such gene is evident in most of the neuroblastoma rapidly progressive forms and predicts a very poor prognosis irrespective of age and clinical stage [74]. Furthermore, this was recorded in 20–25% of primary neuroblastoma cases and about 40% of patients with advanced disease [69].

#### 3.4.2. Deletion or Allelic Loss of Chromosomes 1p and 11q

Neuroblastoma occasionally presents with the unbalanced deletions of the chromosome 1 short arm (1p) and that of chromosome 11 long arm (11q23). This loss of heterozygosity has been linked to less favourable clinical prognosis [74]. Contrasting the 1p deletion, the 11q23 elimination is inversely related to the* MYCN* amplification [75]. Furthermore, conclusions associated the presence of the 1p arm deletion with advanced disease stage [74].

#### 3.4.3. Gain in Chromosome 17q

Chromosomal gain aberrations are also evident in neuroblastoma, such as the 17 long arm gains. This particular unbalanced gain is associated with adverse prognostic factors including stage 4 disease, MYCN amplification, and the mentioned aberration of chromosomes 1p and 11q [67].

#### 3.4.4. DNA Ploidy

Furthermore, the DNA content is sometimes altered with occurrence of triploid and “*near triploid*” accounting for more than half the cases of primary neuroblastoma. The remainder fraction cases are likely to have a defect in genomic stability, resulting in chromosomal rearrangements, unbalanced translocations, and deletions [59, 68].

### 3.5. Treatment

Management of the treatment for neuroblastoma includes the multimodal therapy of surgery, chemotherapy, radiotherapy, and biotherapy [67]. Prior to treatment, the paediatric oncologist classifies the case into a risk group to follow the appropriate treatment. The Children Oncology Group resolved these criteria through “The Risk Stratification System” that incorporates the patient age at diagnosis and INSS stage as mentioned above, as well as tumour histopathology, DNA index, and MYCN gene status [60]. Collectively, these features will aid in assigning the patient under one of these three stages: low, intermediate, or high-risk. Cisplatin, etoposide, doxorubicin, and cyclophosphamide drugs are commonly used in chemotherapy management for neuroblastoma.

## 4. Mechanisms of Chemoresistance by Chemotherapeutic Drugs

Cytotoxic drugs target cells that are actively proliferating, suggesting that they are progressing through the active phases of the cell cycle. Although cytotoxic drugs are effective in multiple cancer models, specific drugs target unique stages in the cell cycle and therefore the choice of drug depends on the molecular features of the cancer condition [76].

A typical example in breast cancer treatment would be a therapy of a HER-2 positive breast cancer, where the aim is to diminish the amplification of the receptor. Alternatively, since BRCA genes play a fundamental role in repairing DNA damage, treating a mutated BRCA malignancy must deal with proteins involved in DNA repair that are not being properly repaired by the BRCA protein products [77]. The following sections focus on describing the two most utilized chemotherapeutic agents for NB chemotherapy, namely, doxorubicin and etoposide.

### 4.1. Chemoresistance

Individual types of cancer have been found to be extremely responsive to treatment at first but then become noncompliant to therapy, a phenomenon that is associated with chemoresistance [78]. This can be considered as the inability of the drug to induce its cytotoxic effect to its maximum potential. Multiple cellular compartments are responsible for this occurrence and various mechanisms can be initiated by individual tumour cells at particular timeframes during the development of cancerous growth [55]. Membrane glycoproteins, tyrosine kinases, and growth factor receptors all contribute to the development of a drug resistant phenotype [79].

Resistance can be classified as either innate or acquired [79]. Innate resistance develops from factors that are inherent with the individual patient, such as genetic mutations within particular genes [79]. As a case in point, breast cancer cases, which commonly have a poor prognosis as a result of treatment ineffectiveness, have been corroborated to HER gene overexpression by Rexer and Arteaga, possibly due to intrinsic mechanisms against HER-2 targeted therapy [80]. On the other hand, acquired chemoresistance progresses during prolonged drug exposure by developing a drug resistant phenotype [79].

Chemoresistance is noted to develop against a plethora of drugs regardless of diverse chemical structures and different mechanisms of intracellular activity, known as multidrug resistance (MDR) [81]. The most fundamental mechanisms causing MDR in tumour cells can be classified into nonclassical MDR and transport-based classical MDR [82]. The nonclassical MDR includes alterations of enzyme systems activity that reduce the targeted activity of the drug [82]. A typical example is glutathione S-transferase, an important enzyme responsible for xenobiotic excretion [82]. Increased activity of this enzyme has been seen in various drug resistant MCF-7 breast cancer cell lines [83, 84]. Another type of nonclassical MDR defect arises from an imbalance of antiapoptotic and proapoptotic proteins causing alterations in cell death signaling pathways and thus inhibition of apoptosis [85]. The classical cellular mechanisms of MDR involve insignificant cellular uptake of the drug [86] and an increase in the efflux of the drug due to an augmentation in the expression of ATP-binding cassette proteins, reducing the drug to insignificant doses [87].

Particularly in breast cancer, the key ABC transporters involved in drug resistance include the P-glycoprotein (PGP), multidrug resistance-associated protein 1 (MRP1), and the breast cancer resistance protein (BCRP) [88]. The PGP was the initial transporter found to be abundantly expressed in breast cancer cell lines exhibiting MDR [89]. PGP acts on a variety of substrates, such as cytotoxic agents in tumour cells [90]. Studies validated that an increase in PGP expression has been found in a substantial amount of breast cancer patients, with a further increase in expression following treatment with chemotherapeutic drugs [90]. PGP is responsible for the transport of both neutral and cationic hydrophobic compounds, including doxorubicin and etoposide [91]. The efflux of substances out of cells is powered by two ATP molecules which fit in PGP's two ATP-binding domains [91].

MRP1 is another transporter protein that is highly expressed in a number of sites including breast tissue [92]. MRP1 also requires two molecules of ATP to actively pump substances out of cells [91]. Likewise, studies have validated that an augmentation in the expression of MRP1 develops resistance to various cytotoxic drugs, including doxorubicin [93].

The breast cancer resistance protein (BRCP) has also been found in a number of malignancies and similar to other proteins, it is linked with chemoresistance to a spectrum of cytotoxic agents, including doxorubicin and etoposide, amongst others [94]. However, unlike the other two transporters, BRCP has only one nucleotide binding domain, meaning that a single molecule of ATP is sufficient to transport substances out of the cells [91].

## 5. miRNAs

The introduction of RNA interference (RNAi) technology in the 1990s gave a new insight into factors affecting gene regulatory processes. Noncoding sequences of the transcriptome have been in fact found to play major roles in gene regulation [95].

Novel research has been linking the behavior of microRNAs (miRNAs) with having a crucial contribution to the development of cancer which have lately been identified as chief regulators of specific genes that are responsible for the mechanisms initiating chemoresistance [96, 97].

MiRNAs are noncoding ribonucleic acids (RNAs) of approximately 22 base pairs in size originating within the cell and function as chief regulators in gene expression [98]. Their role takes place posttranscriptionally by binding with mRNAs within genes, forcing suppression of translation [86].

### 5.1. Mechanism of miRNAs

Primary miRNAs (pri-miRNAs) are the initial transcribed form of miRNAs [99] and are in fact approximately 70 nucleotides long [100]. Pri-miRNAs have a methylated cap at the 5′ end and polyadenylation at the 3′ end [101]. These then form distinctive hairpin-shaped stem-loop secondary structures and associate with a microprocessor complex comprised of Drosha, which is an RNase III endonuclease, and cofactor DGCR8/Pasha, which is a protein having two double-stranded RNA binding domains [102]. Within this complex, these hairpin-shaped pri-miRNAs are processed into 60–70 nucleotide pre-miRNAs and transported into the cytoplasm via Exportin-5 [103, 104]. Once they reach the cytoplasm, pre-miRNAs are converted to a short miRNA duplex by another RNase III endonuclease, Dicer [105]. One strand of this duplex is the final mature miRNA which can then enter the RNA-induced silencing complex (RISC) and guides RISC to the target mRNAs [106].

This complex is directed to the target mRNA via complementary base pairing with the single-strand miRNA comprised within the complex [107]. Once the miRNA is taken up by the RISC, the complex that is formed attaches to the 3′ untranslated region (UTR) of the targeted mRNA, resulting in posttranscriptional gene silencing by impeding translation of the ribosome [108]. The overall effect of this complex results in either (i) translational suppression, (ii) messenger RNA (mRNA) degradation, or (iii) site-specific cleavage [108]. These mechanisms thereby result in gene silencing and are carried out by Argonaute proteins [109]. Two Argonaute (Ago) proteins, Ago 1 and 2 belonging to the Argonaute family of proteins, have been identified as the main catalytic endonucleases in human RISC [109, 110].

Numerous studies have associated dysregulated behavior of miRNAs with a number of conditions, particularly cancer [10, 79, 86, 111–114]. Calin et al. validated that at least half of the miRNA genes are found in cancer-associated genomic regions [115] thereby indicating that miRNAs have a crucial responsibility in the development of a range of malignancies.

### 5.2. Role of miRNAs in Tumourigenesis

Upon assessing the levels of miRNA in tumour tissue against normal tissue, studies established that many of the miRNAs are abnormally expressed in tumour cells, thereby leading to aberrant control and production of its target mRNA [116]. Although not all of the identified miRNAs contribute to the oncogenic phenotype, many of them are found to play a direct role [116, 117]. Their aberrant behavior is apparent in a number of cancers, demonstrating that they can take the role of oncogenic or tumour suppressor miRNAs [116].

If miRNAs are found to be upregulated in cancer cells, significant downregulation of their target genes would result. These target genes can have an impact in both cellular pathways and genes that control the stimulation and progression of a disease. The miRNAs that are upregulated are termed as oncogenic miRNAs (oncomirs) and can target tumour suppressor genes, causing significant downregulation of tumour suppressors, such that their function in maintaining normal cellular control is disrupted [114]. Specifically, Jiang et al. identified the augmented expression of miR-155 in breast cancer cells, where MCF-7 cells were in fact stimulated to grow three times as much [118]. Another typical oncogenic miRNA is miR-21 where its upregulation has been associated with resistant breast cancer cells [10]. The target of miR-21 is the phosphate and tensin homolog (PTEN) tumour suppressor gene which in turn activates the PKI3/AKt pathway that triggers cellular apoptosis [10]. Abnormal silencing of the PTEN gene will result in diminished apoptosis allowing abnormal cellular proliferation [10].

The opposite effect occurs by tumour suppressor miRNAs where their abnormal downregulation results in upregulation of their target oncogenes. The role of oncogenes will be hugely intensified such that the cells receive continuous growth signals, allowing abnormal cell proliferation [114]. One particular tumour suppressor miRNA, miR-205, has been found to be substantially reduced in four different breast cancer cell lines when compared to healthy breast cells [119]. Lorio et al. established that miR-205 targets the receptor of the HER3 oncogene. Downregulation of miR-205 in cancer cells will result in an elevated expression of the HER3 which stimulates aggressive tumour growth [120].

Development and progression of cancer are influenced by whether such key miRNAs are highly or poorly expressed, indicating that a tumor may form due to upregulation of an oncogenic miRNA or the downregulation of a tumour suppressor miRNA or both in tandem.

### 5.3. Role of miRNAs in Tumour Metastasis

Apart from enhancing tumor proliferation, miRNAs are also linked with tumor metastasis, if their target genes are correlated with the metastatic phenotype of cancer cells [121]. Significantly, miR-10b is the prominent miRNA that has been found to have a metastasis-promoting effect [122]. MiR-10b has been identified as the target of an oncoprotein that enables epithelial-mesenchymal transition (EMT), Twist1 [113]. EMT is a crucial process in the metastatic cascade that is denoted by cells acquiring migratory and invasive properties following a loss of cell-cell junctions and cellular polarity [123]. Current studies validated the overexpression of Twist1 in metastatic breast cancer cells, which thereby causes an amplified expression of miR-10b [122]. Han et al. established a link between the stimulation of miR-10b and the TGF-*β*1 signaling pathway, where the overexpression of miR-10b in breast cancer cells and tissues was found to be overexpressed [123]. Furthermore, the level of augmented expression was also found to be highly related to breast cancer aggressiveness [123]. Potential inhibition of miR-10b could serve as an effective target to deter metastasis and treat breast cancer due to the partial inhibition of TGF-*β*1 induced proliferation and EMT [123].

### 5.4. miRNAs Linked with Chemoresistance in Cancer Cells

These short noncoding RNAs emerged as key posttranscriptional regulators of gene expression. Consequently, their dysregulation may lead to abnormal gene expression, seen evidently in the regulation of certain pathways [124].

An underlying cause of multidrug resistance (MDR) is the overexpression of one or more of the ATP binding cassette transporters [79]. Through the work of Bao et al., it was found that miR-298 complemented the 3′UTR of one of these transporters, P-glycoprotein (P-gp) mRNA [125]. Through the same study, chemoresistant developed cell lines expressed a depression in this miRNA level, enhancing even more the evidence that P-pg upregulation is inducing chemoresistance [125]. Moreover, it also concluded that miR-298 is truly responsible for the suppression of P-gp [125]. The latter literature was based on breast cell line; howbeit [126] reported upregulation of miR-27a and miR-451 in two parent cell lines that were also MDR. In such case the miRNAs upregulation was correlated with the increased expression of P-gp and MDR1 mRNA translation [126].

Failure of cells to enter the apoptosis pathway is one of the methods of chemoresistance [127, 128]. In turn, recent studies showed the involvement of miRNAs to regulate apoptosis by the interaction with such mRNAs.

The death-inducing signalling complex (DISC) plays an important role in such regulation of apoptosis pathway [129]. The TRAIL-induced apoptosis pathway that is related to this DISC complex is evidently affected by the level of miRNAs [130]. The miR-221 and miR-222 were found to be upregulated in TRAIL resistant cells while downregulated in sensitive cells [131]. The study revealed that when treating the TRAIL resistant cells with anti-miRNA for mentioned miR-221 and miR-222, the cells regained their sensitivity towards drugs [131].

AKT-dependent pathway regulates cell processed by mediating extracellular and intracellular signals [132, 133]. The Akt pathway potentially acts as antiapoptotic pathway that requires the presence of miR-21 to employ the chemoresistant effect [132]. The relationship was found in glioblastoma cells lines, where the apoptotic process was restored after inhibition of this miR-21 [134].

In another study, miR-200c inhibits the apoptosis induced by Fas-associated phosphatase-1 (FAP-1), acting as an antiapoptotic miRNA [135]. Moreover, evidence even proved that miR-200c is involved in the activation of the mentioned Akt signalling pathway [136].

Conversely, protoapoptotic miRNAs level is significantly lower in apoptotic pathway induced chemoresistance [137]. MiR-34 knockdown has been linked to resistance to apoptosis, with a further link done to the activation of p53 [137, 138]. Furthermore, BCL-2 protein is considered an antiapoptotic protein that is downregulated by miR-34 [139].

Other evidence proved that overexpression of miR-140 causes chemoresistance in human osteosarcoma and colon cancer cells [140]. In both cases, the cell lines were of wild-type p53 [140].

### 5.5. MicroRNA in Neuroblastoma

Studies on neuroblastoma also revealed a pattern of miRNA dysregulation upon certain mutations that consequently lead to chemoresistance [124, 141, 142]. The study by Bray et al. showed evidence that in MYCN amplified neuroblastoma cell lines, seven miRNAs were significantly over- or underexpressed relative to single MYCN copy tumour cells. Secondly, the study also concluded that in large chromosomal imbalances of neuroblastoma cell line, such miRNA dysregulation was involved [141]. Chen and Stallings were able to indicate that miR-184 has a significant role in the apoptosis of neuroblastoma with suggestions that MYCN also mediates tumorigenic effect by regulating the miRNAs [142].

Recently, in 2012 the miR-21 oncomir was identified to have an effect on chemoresistance in neuroblastoma [143]. Cell lines in this study were rendered resistant to cisplatin where the level of expression of miR-21 was proven to be elevated when compared to sensitive cell lines. Chen et al. analyzed this effect through the sensitivity towards drugs that these cells obtain following miR-21 inhibition. In further literature, the MYCN gene was knocked down in neuroblastoma cells, with a subsequence upregulation of the miR-21, concluding an inversely proportional relationship [144].

### 5.6. Association of miRNAs with Breast Cancer

A relatively good amount of miRNAs has been linked with breast cancer in particular (see Table 2). Depending on the gene they target, miRNAs can either affect cellular mechanisms allowing abnormal growth, promote metastasis, or play a crucial role in the development of a drug resistant phenotype. Apart from worsening the prognosis, their abnormal behavior also ceases the effect of therapy, leading to the challenging chemoresistance issue described earlier.

In point of fact, Singh and Mo investigated 76 breast cancer and 10 normal breast samples where 29 miRNAs where found to be abnormally expressed, miR-125b, miR-145, miR-21, and miR-155 being the mostly dysregulated [145].

O'Driscoll and Clynes certified that acquired and intrinsic chemoresistance causes treatment to be unsuccessful in at least one of two breast cancer patients [146]. Considering that aberrant behaviour of miRNAs does play a role in tumourigenesis and development of chemoresistance, stabilizing this aberrant behavior of miRNAs can serve as a therapeutic potential for a wide spread cancer condition like breast cancer.

Developing miRNA-targeted therapeutics depends on the genes the miRNA in question targets, such that miRNA antagonists can be used to regulate gain-of-function miRNAs, whereas miRNA mimics can be used to enhance loss-of-function miRNAs. A miRNA antagonist, anti-miR, is an extensively chemically modified miRNA passenger strand which has a high affinity for the miRNA strand [116]. The binding generated between the two is usually significantly strong such that it is quite irreversible, resulting in a stable miRNA duplex that cannot be processed and thereby degraded by RISC [116]. As a result, extensive silencing of the target gene is reduced.

Conversely, miRNA mimics, miR-mimic, function by triggering gene silencing. miR-mimics are developed by artificial double-stranded miRNA-like RNA fragments [147]. Specifically, this RNA fragment is generated with a free 5′ end which has a partially complementary sequence to the 3′-UTR sequence of the target gene [147]. This artificial miRNA behaves like the endogenous miRNA and specifically binds to the target gene and inhibits translation posttranscriptionally [147]. With reference to the downregulation of miR-451 which is associated with multidrug resistance, a miR-mimic was found to make up for the loss of gene silencing [148]. Kovalchuk et al. validated that transfection of doxorubicin-resistant cells with commercially prepared miR-451 resulted in improved sensitivity to doxorubicin [148]. This signifies that upregulation of the reduced miRNA expression improved the sensitivity of the cells. This crucial principle can serve as a potential method to overcome the highly emerging problem of chemoresistance and limit tumour growth, leading to improvement of the outcome.

## Figures and Tables

**Table 1:** Classification of the different breast cancer subtypes and their most commonly associated molecular features and their related prognosis.

Breast cancer subtype	Molecular features	Clinical outcomes	References
Luminal A	ER+ and/or PR, HER2−	Stage I	[149]
Luminal B	ER+ and/or PR+, HER2+	Stage I (III also reported)	[149, 150]
HER2-enriched	ER− and/or PR−, HER2+	Mostly stage III	[149, 150]
Basal-like subtype or triple-negative	ER− and/or PR−, HER2−, BRCA1	Mostly stage III	[149–151]

ER: estrogen receptor, PR: progesterone receptor, HER: human epidermal growth factor receptor, and BRCA: breast cancer genes; + indicates the presence of the receptor; − indicates the absence of the receptor.

**Table 2:** miRNAs found to be associated with breast cancer and their respective target genes.

miRNA	Dysregulated expression	Target genes	References
miR-10a	Upregulated	Hox genes	[152, 153]
miR-21	Upregulated	PTEN, PDCD4	[154, 155]
miR-29a	Upregulated	PTEN	[155, 156]
miR-125b	Upregulated	E2F3	[155, 157]
miR-203	Upregulated	SOC3	[158]
miR-210	Upregulated	Efna3	[159]
miR-222	Upregulated	PTEN	[155, 157]
miR-30c	Downregulated	TWF I	[160]
miR-31	Downregulated	PKC epsilon	[161]
miR-34a	Downregulated	NOTCHI	[162]
miR-93	Downregulated	TGF*β*R2	[101, 155]
miR-128	Downregulated	BMII, ABCC5	[163]
miR-137	Downregulated	Pgp indirectly	[164]
miR-205	Downregulated	HMGB3	[119, 155]
miR-200a and 200b	Downregulated	ZEB 1/2	[155, 165]
miR-200c	Downregulated	ZEB I, CDH I, PTEN	[155, 166, 167]
miR-298	Downregulated	MDR (P-gp)	[168]
miR-487a	Downregulated	ABCG2 (BRCP)	[169]

PDCD4: programmed cell death 4, E2F3: E2F transcription factor 3, SOC3: suppressor of cytokine signalling 3, Efna3: ephrin A3, TWF I: twinfilin actin-binding protein I, PKC: protein kinase C, TGF*β*R2: transforming growth factor-*β* receptor II, ABCC5: ATP-binding cassette, Subfamily C Member 5, HMGB3: high mobility group box 3, ZEB: zinc finger E-box, CDH: cadherin, and ABCG2: ATP-binding cassette, Subfamily G Member 2.
